# Propofol Reduced Mammosphere Formation of Breast Cancer Stem Cells via PD-L1/Nanog *In Vitro*

**DOI:** 10.1155/2019/9078209

**Published:** 2019-02-14

**Authors:** Xiaobei Zhang, Fangxuan Li, Ying Zheng, Xiaokun Wang, Kaiyuan Wang, Yue Yu, Hongwei Zhao

**Affiliations:** ^1^Department of Anesthesiology, Tianjin Medical University Cancer Institute and Hospital, National Clinical Research Center of Cancer, Tianjin 300060, China; ^2^Key Laboratory of Cancer Prevention and Therapy, Tianjin 300060, China; ^3^Tianjin's Clinical Research Center for Cancer, Tianjin 300060, China; ^4^Department of Cancer Prevention, Tianjin Medical University Cancer Institute and Hospital, National Clinical Research Center of Cancer, Tianjin 300060, China; ^5^The First Department of Breast Cancer, Tianjin Medical University Cancer Institute and Hospital, National Clinical Research Center of Cancer, Tianjin 300060, China; ^6^Key Laboratory of Breast Cancer Prevention and Therapy, Tianjin Medical University, Ministry of Education, Tianjin 300060, China

## Abstract

Several researches revealed that propofol, a hypnotic intravenous anesthesia agent, could inhibit the cancer cell proliferation and tumor formation, which might affect cancer recurrence or metastasis and impact patients' prognosis. Cancer stem cells (CSCs) comprised a tiny fraction of tumor bulk and played a vital role in cancer recurrence and eventual mortality. This study investigates the effect of propofol on breast cancer stem cells (BCSCs) *in vitro* and the underlying molecular mechanisms. Tumor formation of CSCs was measured by mammosphere culture. Cultured BCSCs were exposed to different concentrations and durations of propofol. Cell proliferation and self-renewal capacity were determined by MTT assays. Expressions of PD-L1 and Nanog were measured using western blotting and real-time PCR. We knocked down the PD-L1 expression in MDA-MB-231 cells by lentivirus-mediated RNAi technique, and the mammosphere-forming ability of shControl and shPD-L1 under propofol treatment was examined. Mammosphere culture could enrich BCSCs. Compared with control, cells exposed to propofol for 24 h induced a larger number of mammosphere cells (*P* = 0.0072). Levels of PD-L1 and Nanog were downregulated by propofol. Compared with shControl stem cells, there was no significant difference in the inhibitory effect of propofol on the mammosphere-forming ability of shPD-L1 stem cells which indicated that the inhibition of propofol could disappear in PD-L1 knockdown breast stem cells. Propofol could reduce the mammosphere-forming ability of BCSCs *in vitro*. Mechanism experiments indicated that the inhibition of propofol in mammosphere formation of BCSCs might be mediated through PD-L1, which was important to maintain Nanog.

## 1. Introduction

Accumulating evidences suggested that general anesthetics, including intravenous anesthetics, inhalation anesthetics [1], and opioids [2], could affect cancer cell growth and impact patients' prognosis. Propofol (2,6-disopropylphenol), commonly dubbed as “milk of anesthesia,” is one of the most popular intravenous anesthetic agents in modern medicine, which was used commonly for induction and maintenance of anesthesia, procedural and critical care sedation in children [3, 4]. Recent attention has been drawn to explore the role and mechanism of propofol against cancer progression *in vitro* and *in vivo* [5, 6]. Specifically, the proliferation-inhibiting and apoptosis-inducing properties of propofol in cancer have been studied.

In 2018, the American Cancer Society estimates that 266,120 new cases and 40,920 deaths of breast cancer are projected to occur in the United States [7], which is also the most common cancer and the second leading cancer-related death in females among worldwide [8]. Currently, it is considered that breast cancer is a multifactorial disease with different clones of cancer cells and other cell types such as stromal, immune, or endothelial cells. There is a subpopulation of cancer cells called cancer stem cells (CSCs), defined by two main properties: differentiation and self-renewal [9], contributing to resist the therapy and reinitiate cancer with all its heterogeneity [10, 11]. Recently, due to exciting effect of immunotherapy targeting to immune checkpoint, T-cell inhibitory molecule programed death-ligand 1 (PD-L1), overexpressed in malignant cells including breast cancer cells, could escape from immunological surveillance [12]. Moreover, its crucial role of immune in killing and eliminating cancer cells has been widely acknowledged. Although its mechanism in the immune tolerance has been known and applied in cancer research and clinical treatment, PD-L1 expressed themselves in membrane and cytoplasm of cancer cells intrinsically [13], in which it takes a role of “shield” to prevent tumor cells from catalyzing [14]. Previous studies had confirmed that PD-L1 is expressed in 20% of subgroup of triple-negative breast cancers, and the overexpression of PD-L1 associated with large tumor size, high grade, poor survival, and highly proliferative properties as well as chemo- and radiotherapy resistance [15–17]. Many studies had reported the mutual effect between PD-L1 and epithelial to mesenchymal transition (EMT). EMT was a crucial oncogenic procedure, which also was a vital process in generating CSCs [18]. Thus, when we investigate the role of propofol on breast cancer stem cells (BCSCs), it is necessary to research the effect and mechanism of PD-L1 in mediating CSC capabilities.

Although propofol induces apoptosis and inhibits the invasion of cancer cells both *in vitro* and *in vivo* via different molecular mechanisms [19, 20], we focused on the effect of propofol on BCSCs regulating via PD-L1 signaling pathway. The aim of this study is to examine the mammosphere formation of stem cell with different doses of propofol and thereby determine whether propofol might be advantageous as an anesthetic for surgeries of certain cancers.

## 2. Material and Methods

### 2.1. Cell Culture

The human breast cancer cell lines MCF-7, MDA-MB-231, and SK-BR-3 were obtained from the Cell Bank of Chinese Academy of Sciences (Shanghai, CHN), which were cultured in RPMI-1640 medium supplemented with 10% fetal bovine serum and penicillin/streptomycin dual antibiotics in 25 ml culture flasks at 37°C in a 5% CO_2_ incubator. The culture medium was changed daily, and the cell morphology was observed.

### 2.2. Mammosphere Culture

1 × 10^3^ breast cancer cells were plated in each well of a 6-well ultralow attachment plate (Corning) with 3 ml serum-free mammary epithelial growth medium (MEGM, BioWhittaker), supplemented with B27 (Invitrogen), 20 ng/ml EGF (Invitrogen), and 20 ng/ml bFGF (BD Biosciences). The culture medium was changed weekly.

### 2.3. CSC Proportion by FACS

When the MCF-7, MDA-MB-231, and SK-BR-3 cell number reached 1 × 10^6^, the cells were digested and fully dispersed into a single cell solution. The cells were labeled with ESA-FITC, CD44-APC, and CD24-PE antibodies. It also should be prepared with the 4 controls: (1) cells labeled with 3 isotype-matched control Ab; (2) cells labeled with CD44-APC Ab and 2 other isotype-matched control Ab; (3) cells labeled with ESA-FITCAb and 2 other isotype-matched control Ab; and (4) cells labeled with CD24-PE Ab and 2 other isotype-matched control Ab. The proportion of ESA^+^CD44^+^CD24^-/low^ cells was tested by flow cytometry.

### 2.4. Propofol Treatment

The formulation of propofol was used in this research dissolved in 10% intralipid (the formulation for clinical use, from AstraZeneca). The mammosphere cells of MCF-7 and MDA-MB-231 were grown in 6-well plates, divided into the following two groups: (1) control and (2) propofol (10 *μ*M).

### 2.5. MTT Assay

MTT assay (the 3-(4-5-dimethylthiazol-2-yl)-2, 5-diphenyl tetrazolium bromide dye reduction assay) was performed to compare the effect of propofol in different concentrations of 1 *μ*M, 10 *μ*M, 25 *μ*M, 50 *μ*M, and 100 *μ*M, respectively, or different times of 24 h, 36 h, 48 h, and 72 h. Each condition was replicated in five wells.

Twenty-four hours after treatment, 20 *μ*l of MTT (5 mg/ml in PBS) was added to each well. After 4 h, the supernatant was discarded, and 150 *μ*l of dimethyl sulfoxide (DMSO) was added to each well and mixed by vortexing for 10 min. The optical density (OD) of each well was determined using an ELISA reader, and the drug action curve was plotted.

### 2.6. RNA Extraction and Quantitative Real-Time PCR

Total RNA was extracted from MCF-7 and MDA-MB-231 of different treatments using the TRIzol Reagent (Invitrogen) according to the manufacturer's instructions. A reverse-transcription PCR (RT-PCR) system (TaKaRa) was used. Then, 1 mg sample of the cDNA was quantified by real-time PCR using primer pairs with SYBR Green PCR Master Mix (TaKaRa). Each sample was done in triplicate. *β*-Actin was used as loading control. PCR primers used included PD-L1(5′- TATGGTGGTGCCGACTACAA -3′ and 5′- TGGCTCCCAGAATTACCAAG-3′), Nanog (5′-TTTGTGGGCCTGAAGAAAACT-3′ and 5′-AGGGCTGTCCTGAATAAGCAG-3′) and *β*-actin, an endogenous control (5′-CAGAGCAAGAGAGGCATCC-3′, reverse primer 5′- CTGGGGTGTTGAAGGTC-3′).

### 2.7. Protein Extraction and Western Blot

After drug-treating time came to 24 h, cells were collected for protein extraction. Cells were lysed in RIPA buffer (1% NP-40, 1 mmol/l Na_3_VO_4_, 1 mmol/l NaF, 0.5 mmol/l PMSF) on ice for 30 min. Lysate was abandoned by centrifugation while the supernatant was removed. Protein concentrations were assessed using the BCA Protein Assay Kit (Pierce) and the absorbance was read at 490 nm by means of ELISA reader. Cell lysate containing 30 *μ*g of total protein was run on 10% SDS-PAGE and electrophoretically transferred to polyvinylidene difluoride membranes. The membrane was blocked with 5% blotting grade milk (Bio-Rad) in TBS-T (0.1% Tween 20 in TBS) and then probed with the following primary antibodies: Nanog (Abcam), PD-L1 (Abcam), OCT-4 (CST), SOX-2 (CST), and *β*-actin (CST) at 4°C. Next day, the membrane was incubated with HRP-conjugated secondary antibodies (CST). Fluorescence signal was visualized using SuperSignal West Pico Chemiluminescent Substrate (Pierce).

### 2.8. Plasmids, Lentivirus Production, and Transduction

For knockdown of PD-L1, shRNA plasmid, shControl plasmid, and lentivirial packaging system were purchased from GeneChem (Shanghai, China). Following the manufacturer's instructions of GeneChem, the packaged lentiviruses were harvested for 48 h after shPD-L1/shControl contransfection with lenti-Easy Packaging Mix to infect MDA-MB-231 cells. PD-L1 knockdown was confirmed using real-time PCR and western blot analysis.

### 2.9. Statistics

The measurement data were presented as mean ± S.D. and analyzed with such statistical methods as Student's *t*-test and two-way-ANOVA. The statistical analysis was conducted using the SPSS 17.0 software. The significance level (*α*) was 0.05.

## 3. Results

### 3.1. Mammosphere Culture Enriches BCSCs

In addition to tumor stem cell sorting, mammosphere culture is a very important method to measure the tumor formation of stem cells. Based on the current understanding of CSCs, the scientists believe that noncancer stem cells, placed in serum-free environment and suspension culture, will occur nestling apoptosis phenomenon, which loss the ability to form mammospheres, while CSCs can withstand nested apoptosis to form spherical structures of microspheres by unlimited self-renewing.

**Figure 1:**
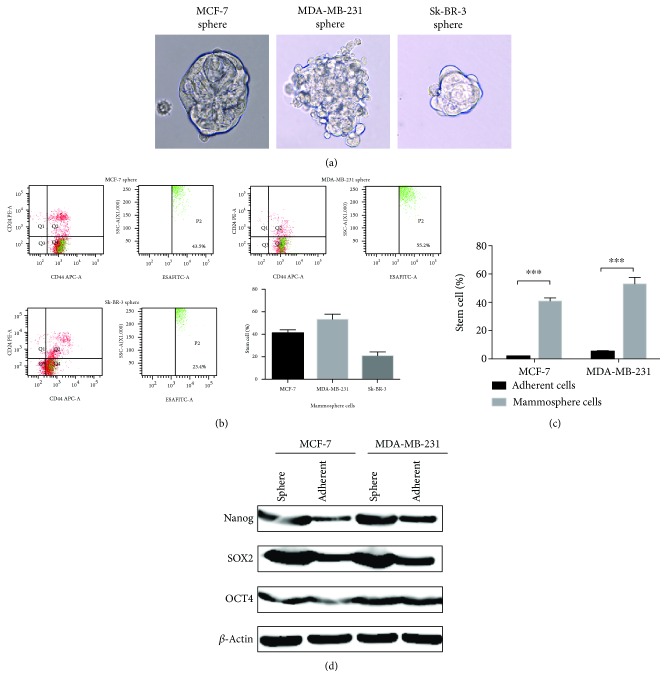
Mammosphere culture could enrich BCSCs. (a) The microstructure of mammospheres of different cell lines. (b) The percentage of ESA^+^CD44^+^CD24^-/low^ in mammosphere of different cell lines. (c) The different percentage of ESA^+^CD44^+^CD24^-/low^ in adherent cells and mammosphere cells. (d) The expression of stem cell-associated proteins Nanog, OCT4, and SOX2 proteins in normal breast cancer cell lines and microspheres. Each column represents the mean ± S.D (*n* = 3). The statistical analysis was performed with Student's *t*-test. ^∗∗∗^ *P* < 0.001.

In our study, the microtubule formation ability of different breast cancer cell lines was different: the ductal carcinoma cell line MCF-7 could form a typical spherical structure, while the HER-2 overexpression cell line SK-BR-3 and TNBC cell line MDA-MB-231 could not form a typical spherical structure, replaced by a loose group, bead-like structure (Figure 1(a)), which suggest that the morphology of microspheres might be different in different cell lines. In the same cell line, the formation of microspheres is a reflection of the characteristics of stem cells, but different cell lines could not use only microsphere morphology to determine the strength of stem cells.

In order to assess the stability and reliability of the breast cancer mammosphere culture system, we analyzed the mammosphere proportion of BCSCs in different cell lines by FACS. The percentage of ESA^+^CD44^+^CD24^-/low^ in MCF-7 mammosphere was 40.7%±2.59%, the ratio of MDA-MB-231 was 52.73%±5.25%, and the ratio of SK-BR-3 was 20.57% ± 3.76% (Figures 1(b) and 1(c)), showing that microsphere culture of different cells can enrich the BCSCs.

Malignant cells reinitiate tumors relying on self-renewal potency, capacity to proliferate indefinitely, and tolerance to genotoxic stress including chemotherapy and radiation. Series of molecular mechanisms are involved synergistically in inducing the self-renewal proficiency such as embryonic antigens (Nanog, OCT-4A, and SOX-2) and the dysregulation of Notch, WNT, or Hedgehog self-renewal pathways as well as facilitation of chromatin regulators. Subsequently, we compared the expression of stem cell-associated proteins Nanog, OCT4, and SOX2 proteins in normal breast cancer cell lines and microspheres. The expression of Nanog, OCT4, and SOX2 in microsphere cells was higher than that in normal cells (Figure 1(d)). These results suggested that microsphere culture could enrich BCSCs.

### 3.2. PD-L1 Was Overexpressed in Mammosphere Cells of Breast Cancer

**Figure 2:**
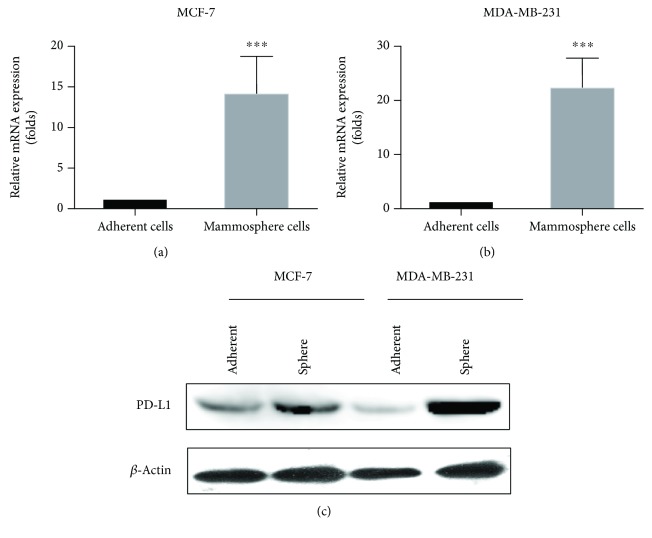
PD-L1 was overexpression in mammosphere cells of breast cancer. (a) Compared with adherent cells, the expression of PD-L1 mRNA in mammosphere of MCF-7 was increased. (b) Compared with adherent cells, the expression of PD-L1 mRNA in mammosphere of MDA-MB-231 was overexpressed. (c) The protein level of PD-L1 in BCSCs derived from microspheres significantly increased compared with adherent cells. ^∗∗∗^ *P* < 0.001; compared with the adherent cells.

Some researchers believed that PD-L1 was mainly expressed in a subset of hormone-negative breast cancer patients and its expression correlated with bad prognostic markers, which was associated with highly proliferating cells and contributes to chemoresistance. We analyzed the expression of PD-L1 in BCSC and found that the expression of PD-L1 mRNA (Figures 2(a) and 2(b)) and protein (Figure 2(c)) was significantly increased in BCSCs derived from microspheres compared the adherent cells, suggesting that PD-L1 may have an important effect on the stem-characteristic maintenance of BCSCs.

### 3.3. Propofol Reduced the Mammosphere Formation of Breast Cancer and Downregulated the Expression of PD-L1 and Nanog

**Figure 3:**
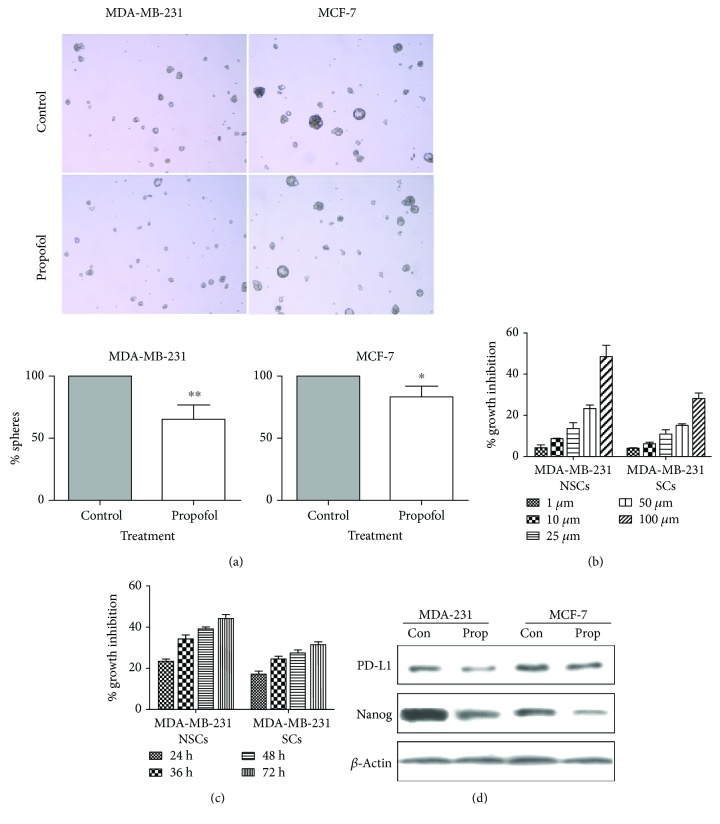
Propofol reduced the mammosphere formation of breast cancer and downregulated the expression of PD-L1 and Nanog. (a) Mammosphere formation ability of propofol group was significantly inhibited compared with the control group (*P* = 0.0072). (b) Propofol could inhibit the proliferation of breast cancer cells and BCSCs in concentration dependent. (c) Propofol could inhibit the proliferation of breast cancer cells and BCSCs in time dependent. (d) The expression of PD-L1 and Nanog in different types of stem cells was detected by western blot. ^∗^ *P* ≤ 0.05; ^∗∗^ *P* < 0.01; compared with the control group.

Non-stem cells (NSCs) and stem cells (SCs) of MCF-7 and MDA-MB-231 were cultured with propofol for 24 h in concentration of 1 *μ*M, 10 *μ*M, 25 *μ*M, 50 *μ*M, and 100 *μ*M, respectively. The results showed that propofol could inhibit the proliferation of breast cancer cells and BCSCs. However, the inhibitory effect of propofol on breast cancer cells was more sensitive (*P* < 0.01) (Figure 3(b)). Then, we tested the inhibited effect of propofol on NSCs and SCs for different times (24 h, 36 h, 48 h, and 72 h), showing that propofol could inhibit the proliferation of NSCs and SCs in time dependent (Figure 3(c)).

Propofol (10 *μ*M) was administered to the mammosphere cells of MDA-MD-231 and MCF-7, respectively. It was found that the mammosphere formation ability of propofol group was significantly inhibited compared with the control group (*P* = 0.0072). In MCF-7 stem cells, propofol also inhibited its mammosphere-forming ability (*P* = 0.0307), suggesting that propofol can effectively inhibit the mammosphere formation of BCSCs (Figure 3(a)).

The expression of PD-L1 and Nanog in different types of stem cells was detected by western blot. It was found that under the action of propofol in BCSCs of MDA-MB-231 or MCF-7, the expression of both PD-L1 and Nanog was downregulated (Figure 3(d)).

### 3.4. Propofol Could Not Reduce the Mammosphere Formation of shPD-L1 Cells In Vitro

**Figure 4:**
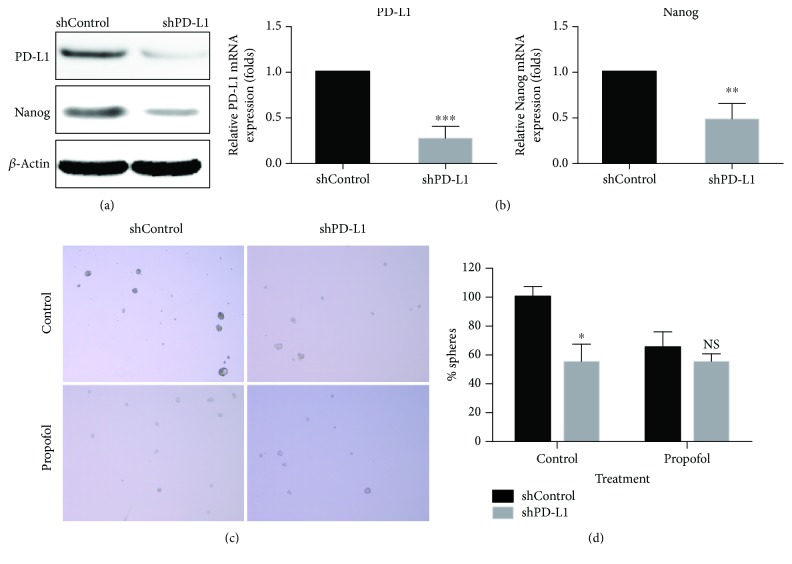
Propofol could not reduce the mammosphere formation of shPD-L1 cells *in vitro*. (a) LV-shPD-L1 significantly reduced the protein expression of PD-L1 and Nanog in cells by western blot. (b) LV-shPD-L1 significantly reduced the expression of PD-L1 and Nanog mRNA in cells by real-time PCR. (c, d) Compared with shControl stem cells, there was no significant difference in the inhibitory effect of propofol on the mammosphere-forming ability of shPD-L1 stem cells (*P* > 0.05). NS: not significant; ^∗^ *P* ≤ 0.05; ^∗∗^ *P* < 0.01; ^∗∗∗^ *P* < 0.001; compared with the control group.

In order to investigate the effect of PD-L1 on the stem maintenance of breast cancer cells, we knocked down the PD-L1 expression in MDA-MB-231 cells by lentivirus-mediated RNAi technique. The cells were observed on the third day after being infected with lentivirus, and the infection efficiency was above 90%. We found that LV-shPD-L1 significantly reduced the expression of PD-L1 and Nanog in cells by real-time PCR and western blot detection (Figures 4(a) and 4(b)).

Compared with shControl stem cells, there was no significant difference in the inhibitory effect of propofol on the mammosphere-forming ability of shPD-L1 stem cells (*P* > 0.05) (Figures 4(c) and 4(d)). This result confirmed that propofol could regulate the mammosphere-forming ability of BCSCs through PD-L1.

## 4. Discussion

Many studies have demonstrated the presence of minimal number of CSCs in tumor cells. CSCs not only has the ability to differentiate into various types of tumor cells but also has a long-term self-renewal capability that determines it play an extremely important role in the occurrence and development of malignant tumors [21, 22]. BCSCs have a high survival rate under the action of chemotherapy drugs compared to breast cancer cell lines [23]. Therefore, effective removal of CSCs is essential for achieving the desired anticancer efficacy.

Our previous studies show that ESA^+^CD44^+^CD24^-/low^ breast cancer cells have stem-like cell characteristics and found that BCSCs on the conventional chemotherapy drug docetaxel, endocrine therapy drugs such as letrozole and targeted therapy trastuzumab, have a certain resistance [24–26]. Shi et al. [27] exposed glial CSCs to 2% sevoflurane for 6 hours and found that cell differentiation was also increased; HIF-1*α* and HIF-2*α* were upregulated. HIF siRNA decreased the percentage of proliferating glial CSCs after sevoflurane exposure, which confirmed that sevoflurane could promote the differentiation of glioma stem cells. Sun et al. [28] have shown that sevoflurane affects cells survival and migration ability by regulating H/SD in bone marrow mesenchymal stem cells and upregulates expression of HIF-1*α*, HIF-2*α*, VEGF, and p-Akt/Akt. The effect of other narcotic drugs on CSCs is not clear and requires further research and exploration. In our research, we found that propofol could inhibit the proliferation of breast cancer cells and BCSCs. However, the inhibitory effect of propofol on breast cancer cells was more sensitive (*P* < 0.01). And propofol can effectively inhibit the mammosphere formation of BCSCs.

PD-L1 is an important and hot immune checkpoint in immune research which the effect and mechanism in immune regulating have been well recognized. Recent literatures had evidenced that PD-L1 also played an important role in cancer progression via moderating cancer cells themselves [29–31]. Accumulating evidences had confirmed the correlation between the CSC properties and PD-L1 overexpression [14, 18]. Thus, it suggested us to delve into the mechanism of PD-L1 in influencing the effect of propofol on BCSCs. Almozyan et al. [32] had found that the overexpression of PD-L1 took a direct way to maintain BCSC properties in breast carcinoma. *In vitro* study, PD-L1 promoted continued expression of stemness biomarkers Nanog and OCT-4A by PI3K/AKT pathway. Owing to its impact on the BCSCs, it suggested that anti-PD-L1 therapy could assist the comprehensive treatment of advanced breast cancer and improve its prognosis [32].

Gupta et al. [33] found that silencing PD-L1 in B16 and ID8agg cells by shRNA reduced the canonical tumor-initiating cell (TIC) genes Nanog. In our research, under the action of propofol in BCSCs of MDA-MB-231 or MCF-7, the expressions of both PD-L1 and Nanog were downregulated, and compared with shControl stem cells, there was no significant difference in the inhibitory effect of propofol on the mammosphere-forming ability of shPD-L1 stem cells.

Homologous domain protein Nanog is a key factor in recent years to discover the pluripotency and self-renewal of embryonic stem cells [34], which is considered to be a “master switch” of stem cells that have the ability to develop into various types of cells. Recent studies [34–37] found that in malignant tumors, Nanog expression and tumor stem cell marker expression is closely related. Functional studies have shown that Nanog not only promotes the ability of self-renewal and long-term proliferation of CSCs but also mediates oncogenes that can influence the clinical features and prognosis of malignant patients. In our research, under the action of propofol in BCSCs of MDA-MB-231 or MCF-7, the expression of Nanog was downregulated, which confirmed that propofol could regulate the mammosphere-forming ability of BCSCs through Nanog.

In conclusion, we have observed close association between PD-L1 expression and breast cancer stemness in the breast cancer cell lines. Our work confirmed this inhibitory role of propofol in maintaining breast cancer stemness *in vitro*. Our research has shown that the effect of propofol in CSCs is mediated through PD-L1, which in turn is important to maintain Nanog. Our findings suggest that propofol could affect the mammosphere formation of breast CSCs by targeting PD-L1.

## Data Availability

All data generated or analyzed during this study are included in this published article.
